# Cognitive Dysfunction in Repeat Expansion Diseases: A Review

**DOI:** 10.3389/fnagi.2022.841711

**Published:** 2022-04-11

**Authors:** Sizhe Zhang, Lu Shen, Bin Jiao

**Affiliations:** ^1^Department of Neurology, Xiangya Hospital, Central South University, Changsha, China; ^2^National Clinical Research Center for Geriatric Disorders, Central South University, Changsha, China; ^3^Engineering Research Center of Hunan Province in Cognitive Impairment Disorders, Central South University, Changsha, China; ^4^Hunan International Scientific and Technological Cooperation Base of Neurodegenerative and Neurogenetic Diseases, Changsha, China; ^5^Key Laboratory of Hunan Province in Neurodegenerative Disorders, Central South University, Changsha, China

**Keywords:** cognitive dysfunction, repeat expansion diseases, NIID, C9FTD, HD, FXTAS, SCAs

## Abstract

With the development of the sequencing technique, more than 40 repeat expansion diseases (REDs) have been identified during the past two decades. Moreover, the clinical features of these diseases show some commonality, and the nervous system, especially the cognitive function was affected in part by these diseases. However, the specific cognitive domains impaired in different diseases were inconsistent. Here, we survey literature on the cognitive consequences of the following disorders presenting cognitive dysfunction and summarizing the pathogenic genes, epidemiology, and different domains affected by these diseases. We found that the cognitive domains affected in neuronal intranuclear inclusion disease (NIID) were widespread including the executive function, memory, information processing speed, attention, visuospatial function, and language. Patients with C9ORF72-frontotemporal dementia (FTD) showed impairment in executive function, memory, language, and visuospatial function. While in Huntington's disease (HD), the executive function, memory, and information processing speed were affected, in the fragile X-associated tremor/ataxia syndrome (FXTAS), executive function, memory, information processing speed, and attention were impaired. Moreover, the spinocerebellar ataxias showed broad damage in almost all the cognitive domains except for the relatively intact language ability. Some other diseases with relatively rare clinical data also indicated cognitive dysfunction, such as myotonic dystrophy type 1 (DM1), progressive myoclonus epilepsy (PME), Friedreich ataxia (FRDA), Huntington disease like-2 (HDL2), and cerebellar ataxia, neuropathy, vestibular areflexia syndrome (CANVAS). We drew a cognitive function landscape of the related REDs that might provide an aspect for differential diagnosis through cognitive domains and effective non-specific interventions for these diseases.

## Introduction

Cognitive dysfunction is observed in various neurodegenerative diseases, such as Alzheimer's disease (AD), frontotemporal dementia (FTD), Dementia with Lewy Bodies (DLB), and Parkinson's Disease (PD). Generally, cognitive dysfunction can be divided into several cognitive domains including general intelligence, executive function, memory, attention, psychomotor speed, visuospatial function, and language. All of these domains can be quantified by related scales or tests (Supplementary Table 1). General intelligence is measured through the Wechsler Adult Intelligence Scale (WAIS) and Mini-Mental State Examination (MMSE), and executive function contains several parts like verbal fluency, inhibitory control, and planning. Furthermore, the memory domain is more complex and is composed of long-term and short-term memory. Episodic memory is a part of long-term memory, while working memory, verbal memory, and visuospatial memory are recognized as short-term memories.

In recent decades, since the first discovery of the pathogenic gene of fragile X syndrome (FXS) and expanded CGG repeats in the 5′UTR of fragile X mental retardation 1 (FMR1) gene, more than 40 repeat expansion diseases (REDs) have been discovered, most of which primarily affect the nervous system. The clinical features of these diseases show some general patterns (Paulson, 2018). For example, the expansion size is unstable and often changes when transmitted to the next generation, which causes clinical anticipation; moreover, the clinical manifestation of these diseases is highly variable, and the cognitive dysfunction weighs much in these different diseases, especially the neuronal intranuclear inclusion disease (NIID), C9ORF72-frontotemporal dementia (C9FTD), Huntington's disease (HD), fragile X-associated tremor/ataxia syndrome (FXTAS), and spinocerebellar ataxias (SCAs). Some other diseases, such as myotonic dystrophy type 1 (DM1), progressive myoclonus epilepsy (PME), Friedreich ataxia (FRDA), Huntington disease like-2 (HDL2), and cerebellar ataxia, neuropathy, vestibular areflexia syndrome (CANVAS), have relatively little data and also reveal cognition dysfunction. Altogether, this review will present the introduction, pathogenic gene, epidemiology, and impaired cognitive domains for the different REDs separately.

## Neuronal Intranuclear Inclusion Disease

### Introduction

Neuronal intranuclear inclusion disease (NIID) was first reported in 1968 (Lindenberg et al., 1968). It is a disease with great clinical heterogeneity, and often presents with muscle weakness, parkinsonism, dementia, and many other phenotypes (Tian et al., 2019). It is a neurodegenerative disease characterized by eosinophilic hyaline intranuclear inclusions in the central nervous system (CNS) and peripheral nervous system, and also in the visceral organs (Sone et al., 2016).

### Pathogenic Gene

**Figure 1:**
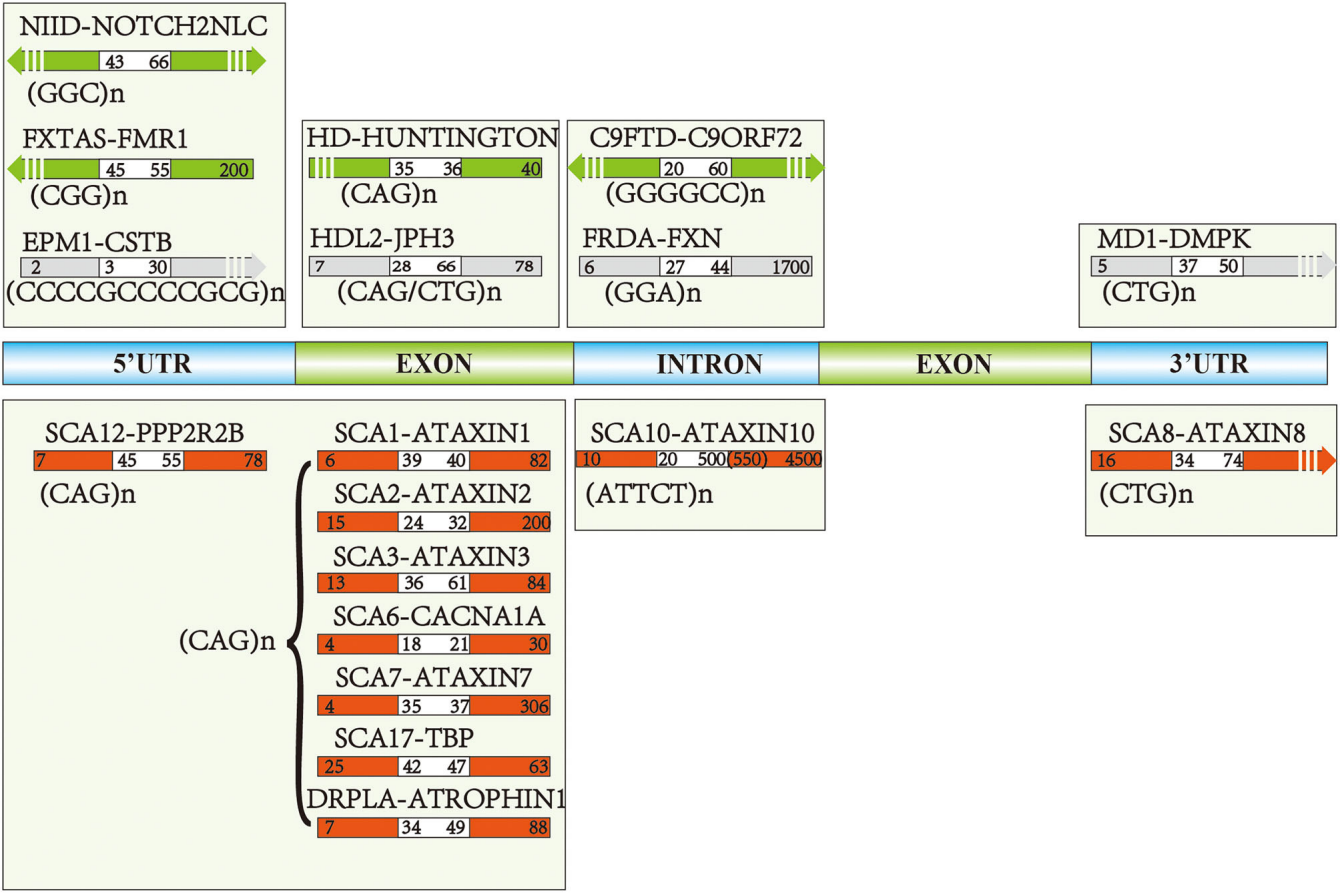
The pathogenic genes and repeat numbers of each disease: green and red arrows reflected pathogenic repeats of the most common repeat expansion diseases (REDs), the gray arrows indicate the diseases with relatively fewer studies focusing on the cognitive aspects. White areas of each arrow represent the intermediate repeats, and the right repeat numbers are pathogenic toward each disease, while left intervals indicate normal repeat numbers.

The genetic background of NIID was not confirmed until 2019. Several teams described a trinucleotide (GGC)n expanded repeats in the 5'UTR region of Notch 2 N-terminal like C (NOTCH2NLC) gene using a long-read genome sequencing (LRS) approach (Figure 1) (Sone et al., 2019; Tian et al., 2019). The normal size of (GGC)n expanded repeats is <43 repeats (Ishiura et al., 2019), and the pathogenic size is above 66 (Tian et al., 2019) or 60 (Sun et al., 2020). Interestingly, one special phenomenon reported by Sone et al. (2019) was that the weakness-dominant phenotype had the (GGC)n followed by varieties of ((GGA)n(GGC)n)n repeats, whereas cases presented with dementia were characterized by pure (GGC)n repeats without interruption).

### Epidemiology

The morbidity of NIID is unclear with relatively rare data, but the prevalence is relatively high in Japan and China, with the most cases reported in these two countries. The age at the onset of NIID dementia subtype varies from the third to 7th decades greatly, and the disease duration generally occurs in those aged 7.6–12.4 years on average. Female patients seem to be affected more than male patients (Sone et al., 2016; Tian et al., 2019).

### Impaired Cognitive Domains

**Figure 2:**
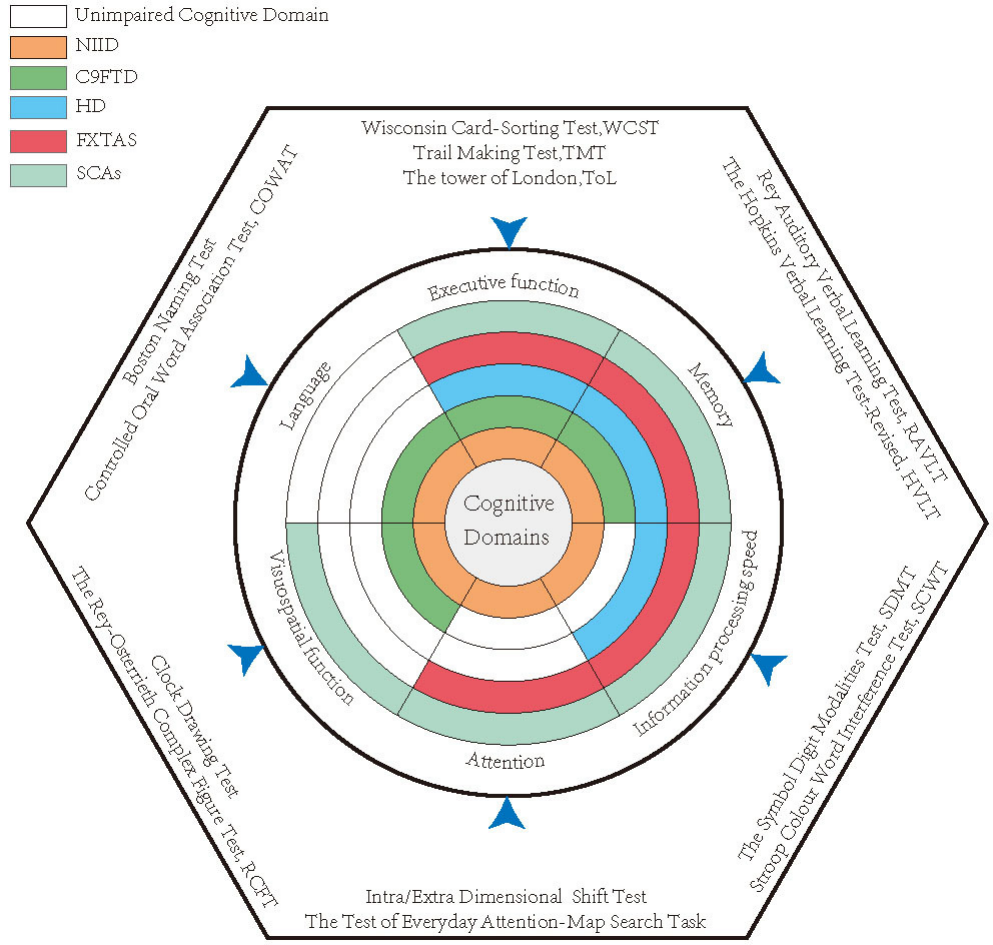
The impaired cognitive domains of each disease and the most classical quantitative scales used in studies. Each annulus represented a disease, and the colored areas indicate the impaired cognitive domains, while the white zones reveal relatively intact cognitive domains.

Dementia is an important symptom both in sporadic cases and in familial cases (Sone et al., 2016). The patients with dementia-dominant NIID could present with memory impairment, disorientation, visuospatial dysfunction, language problem, and execution dysfunction, as well as abnormal behaviors (Figure 2) (Weidenheim and Dickson, 1995; Sone et al., 2014; Yamaguchi et al., 2018; Yadav et al., 2019). These to some extent clinically overlap with AD or other types of neurodegenerative dementia, like FTD (Jiao et al., 2020).

#### Executive Function

One typical characteristic of NIID is severe damage to executive function, in line with the symptoms of subcortical dementia (Huber and Paulson, 1985). In detail, the MMSE is often used for the evaluation of cortical function, but it is heavily weighted toward language, and relatively insensitive to the executive dysfunction of subcortical dementia (Filley, 2012). Other tests including Montreal Cognitive Assessment (MoCA), Frontal Assessment Battery (FAB), and Clock Drawing Test (CDT) have been found to be sensitive to white matter dysfunction (Filley, 2012). FAB is a battery that consists of six subtests exploring the following: conceptualization, mental flexibility, motor programming, sensitivity to interference, inhibitory control, and environmental autonomy (Dubois et al., 2000). The CDT requires participants to draw all the numbers of a clock face on a sheet of paper and move the hands to “10 past 11”, reflecting the impairment of the executive function (Kim et al., 2009). An interesting finding from a study by Sone et al. (2016) showed that the FAB scores declined more obviously than the MMSE scores, particularly in the dementia-dominant group, whether they were sporadic or not. The decline in the FAB score of NIID cases may reflect the white matter damage, and this pattern of decline is similar to other neurological diseases with white matter damage, such as cerebral autosomal dominant arteriopathy with subcortical infarcts and leukoencephalopathy (CADASIL) (Filley et al., 1999; Chabriat et al., 2009) and FXTAS (Kasuga et al., 2011). Recently, Wang et al. (2020) investigated the executive dysfunction in patients with NIID reflected by Trail Making Test (TMT, For Trail A, the participants were required to connect, as quickly as possible, circles containing numbers in the ascending numerical order. For Trail B, the participants were to connect, as quickly as possible, circles containing numbers and letters, alternating between numbers and letters in the ascending order) and CDT. Interestingly, the researchers also found that Fazekas scores were significantly correlated with the global cognition, executive, and language functions (Wang et al., 2020). Researchers used some other scales, such as Addenbrooke's Cognitive Examination Revised (ACE-R), to also demonstrate the executive dysfunction of the patients with NIID (Araki et al., 2016).

#### Other Impaired Domains

One systematic investigation showed that memory, language, and visuospatial function were impaired in patients with NIID (Wang et al., 2020). Bad performance in the Auditory Verbal Learning Test [AVLT, a word learning task in which a list of 15 common, unrelated nouns is read to the patient five times and the patient recalls in any order as many of the words as possible after each reading (Miller et al., 1988)] indicated that memory was impaired in patients with NIID, and the impaired language ability was reflected by animal fluency and Boston Naming Test; Pentagon Copying in MMSE and Cube Copying in MoCA Test indicated that visuospatial function was also impaired (Wang et al., 2020). Other clinical cases reporting cognitive dysfunction or dementia are summarized here. One case with a 5-year history of word-finding problems as the most prominent feature was reported, and the neuropsychological examination also revealed deficits in attention and word fluency, and lower scores in tests exploring information processing, executive function, and memory, but the details of the tests were unclear (Cupidi et al., 2019). Another case presented with difficulty in communication, along with apathy, disorientation, impaired attention, memory disturbance, construction disturbance, and limb-kinetic apraxia; the MMSE score was 17/30, and the subject could not conduct the TMT; moreover, the FAB score decreased to 5/18 after a 5-year follow-up (Kawarabayashi et al., 2018). Furthermore, a case admitted to a hospital with subacute progressive unconsciousness also showed severe cognitive impairment, including apathy, disorientation, and impaired word fluency (Yokoi et al., 2016).

This section has summarized the cognitive domains affected in NIID. Besides executive dysfunction, including language, memory, information processing speed, attention, and disorientation were impaired, it is necessary to confirm these with further research.

## C9ORF72-Frontotemporal Dementia

### Introduction

Frontotemporal dementia (FTD) is a heterogeneous group of syndrome with degeneration of the frontal and temporal lobes (Neary et al., 1998). It is characterized by progressive deterioration in behavior, speech production, or language, with relative sparing of memory and visuospatial function. The FTD can be subdivided into three clinical subtypes: behavioral-variant FTD (bvFTD), progressive non-fluent aphasia (PNFA), and semantic dementia (SD) (Neary et al., 1998). In broad terms, 40–50% of people with FTD have family histories of dementia and related disorders (See et al., 2010). Mutations in *C9ORF72, MAPT*, and *GRN* genes account for about 60% of all cases of inherited frontotemporal lobar degeneration (Bang et al., 2015).

### Pathogenic Gene

The gene, *C9ORF72*, first discovered in 2011, was found to exist as expanded GGGGCC hexanucleotide repeats in the intron1 between exons 1a and 1b. It is the pathogenic gene leading to FTD, motor neuron disease (MND), or both (FTD-MND) (DeJesus-Hernandez et al., 2011; Renton et al., 2011), and it is the most common genetic cause of FTD and amyotrophic lateral sclerosis (DeJesus-Hernandez et al., 2011; Bang et al., 2015). The normal repeat numbers usually are <20 (DeJesus-Hernandez et al., 2011), but there are some people with normal cognition showing 30 repeats in the *C9ORF72* gene (Dobson-Stone et al., 2012). Sometimes it can be over 4,000 repeats in affected people (Bennion Callister and Pickering-Brown, 2014), and the relationship between the repeat size and clinical phenotypes, such as age at onset, disease duration, or clinical severity is unclear (Van Mossevelde et al., 2017).

### Epidemiology

The disorder is the third most common form of dementia across all age groups, after AD and DLB (Van Mossevelde et al., 2018). The pathogenic mutation was first confirmed in Finnish (Renton et al., 2011) and North American subjects (DeJesus-Hernandez et al., 2011), showing a great proportion of mutation carriers in the two areas. After that, the mutation spectrum of *C9ORF72* was evaluated in many European countries; a meta-analysis showed that the frequency of the *C9ORF72* expansions in Western Europe was 9.98% in the overall FTLD, with 18.52% in familial, and 6.26% in sporadic patients with FTLD, and the *C9ORF72* carriers developed dementia at an average age of 55.3 years old, significantly earlier than GRN mutation carriers (van der Zee et al., 2013). However, in Asian countries, such as China (Tang et al., 2016) and Japan (Ogaki et al., 2013), the positive rate is extremely lower than in Western Europe and North America.

### Impaired Cognitive Domains

The *C9ORF72* mutation was associated more frequently with the behavioral variant FTD (bvFTD) (Sha et al., 2012; Simón-Sánchez et al., 2012; Whitwell et al., 2012; Cooper-Knock et al., 2015; Rohrer et al., 2015). Expansion carriers with bvFTD often presented with inappropriate behavior and agitation (Van Langenhove et al., 2013); this was in contrast to patients with bvFTD having a GRN mutation, in whom apathy dominated the clinical picture (Beck et al., 2008; Pickering-Brown et al., 2008). As for cognitive dysfunction, several studies used different neuropsychological tests for the measurement of the different cognitive domains of C9FTD. The cognitive impairment was involved in executive function, memory, attention, naming, calculation, and visuospatial skills.

#### General Intelligence

The verbal and performance IQ of patients with C9FTD were lower than that of healthy people at the baseline (Mahoney et al., 2012a,b), and then became impaired during the follow-up (Mahoney et al., 2012b). The MMSE scores of 12 subjects with C9 mutation showed an average score of 20.5, and 7 patients obtained a score of <24/30, indicating a wide range of cognitive impairment of patients with C9FTD (Boeve et al., 2012). There was also a trend for a greater annualized decline in the MMSE scores in C9 FTLD compared to C9 non-carriers, though no statistical significance was observed (Irwin et al., 2013). Dementia rating scale-2 scores showed similar results as well (Boeve et al., 2012).

#### Executive Function

Neuropsychological evaluation of patients with C9FTD usually demonstrated a remarkable impairment on frontal executive tasks (Mahoney et al., 2012a; Van Langenhove et al., 2013). One study showed that the executive function was severely impaired in the majority of subjects at the baseline (7/12 subjects), and became more frequent over the period of follow-up (10/12), reflected by the impairment in the Stroop Color-Word Test [SCWT, a page with 100 color words (blue, green, and red) printed in non-matching colors was shown to the participants, who were asked to name the colors disregarding the verbal content of the words] or the Hayling Sentence Completion Test (a test consisting of two parts in which the participants are asked to complete a series of sentences) (Mahoney et al., 2012b). Tested subjects presented predominant impairment across timed tasks, such as TMT and Digit Span Test (The participants were required to repeat a series of digits that became gradually longer. The maximum digit span that the participants were able to repeat in direct and reverse orders constituted the forward and backward scores, respectively) (Boeve et al., 2012). Although the impairment of the executive function of C9FTD was obvious, performance on the executive tests, like the verbal fluency test failed to discriminate between *C9ORF72* mutation carriers and non-carriers (Devenney et al., 2014). But another research showed that the C9-FTLD showed a significantly greater annualized rate of decline in letter fluency (4.5 ± 1.3 words/year) than the C9 non-carriers (Irwin et al., 2013).

#### Memory

Patients can also have trouble with memory, including working memory, recognition memory, and episodic memory. The C9bvFTD group presented more impaired working memory than the non-carriers (Sha et al., 2012). The situation was similar in recognition memory, which was composed of verbal memory and visual memory, reflected by the deficit of the Recognition Memory Test for words and faces separately (Mahoney et al., 2012b) [Recognition Memory Test consists of 50 words and 50 photographs of male faces printed individually on white cards and a set of two-choice recognition cards; the patients are allowed 3 s to memorize each word or face, and the memory retention is tested by a two-choice recognition immediately after the presentation of words and photographs. Patients are required to indicate the correct word or face from the paired similar word or photograph (Ho et al., 1990)]. Moreover, the episodic memory impairment was demonstrated by the recall component of the Rey-Osterrieth Complex Figure Test (RCFT, a neurocognitive evaluation in which the individuals are asked to copy a figure and then draw the figure from memory) (Devenney et al., 2014). Similarly, it failed to discriminate the C9FTD from the non-carriers.

#### Visuospatial Function

The visuospatial function was impaired in the C9FTD as well. The results of the visuospatial functioning tests, including block design and judgment of line orientation, showed impairment of visuospatial function, but there were some slight differences between the results of these two tests. Most patients got lower scores on Block Design (Block Design is a timed task that requires motor manipulation of blocks to match target designs and relies on processing speed, visuospatial skills, and an executive problem-solving component.) compared to the judgment of line orientation, which indicated that the higher rate of impairment detected by the Block Design may be partly due to slowed processing speed and executive dysfunction, and not due to visuospatial deficits (Boeve et al., 2012). Another study indicated that the impairment of visual perceptual functions remained largely stable over the period of follow-up, elucidated by the Visual Object and Spatial Perception Battery (VOSP consists of three subsets, dot counting, position discrimination, and cube analysis; the dot-counting VOSP subtask requires the patient to count the number of dots in each square without pointing. The position discrimination VOSP subtask asks the participants to discriminate between squares with either centered or off-centered dots. The cube analysis if VOSP subtask presents a two-dimensional image of several stacked cubes and the patient must determine how many cubes are shown) (Mahoney et al., 2012b).

#### Language

Language problems also existed in subjects with C9FTD. Naming was impaired in one-half of the patients at the baseline(6/12); During follow-up, naming deficits were evident in the majority (8/12), and it was assessed by the Graded Naming Test or the Oldfield Naming Test (Mahoney et al., 2012b). Some other tests, including Boston Naming Test (BNT), Controlled Oral Word Association Test [COWAT, an executive control measure assessing semantic and phonetic cues, where the participants recalled words beginning with the letter A, F, and S within 60 s (Hlubocky et al., 2018)] and semantic/category fluency for animals, fruits, and vegetables, also indicated language problems in patients with C9FTD, though not as common as the executive dysfunction (Boeve et al., 2012). Another study showed that anomia appeared in 47% of the patients with C9FTD (Mahoney et al., 2012a).

## Huntington's Disease

### Introduction

Huntington's disease, first described by George Huntington in 1872, is an autosomal-dominant, progressive neurodegenerative disorder with a distinct phenotype, including hereditary chorea and a distinct phenotype including cognitive and psychiatric impairment (Walker, 2007; Burgunder, 2014).

### Pathogenic Gene

The HD pathogenic mutation, an expanded (CAG)n repeats in exon1 of Huntington (HTT, also named as IT15) gene, was discovered in 1993, leading to a polyglutamine strand of variable length at the N-terminus of HTT protein (MacDonald et al., 1993). The average repeat number of normal people is around 17, and patients with HD have more than 36 repeats, but those with 36–39 have a less severe phenotype owing to a decreased penetrance of the mutation at these allele sizes (Losekoot et al., 2013), and the patients having ≥40 repeats are fully penetrant (MacDonald et al., 1993). Generally, the symptoms appear during the patients' middle age, in their fourth decades (Roos, 2010). The age of the clinical onset, the rate of disease progression, and disease severity are partially determined by the number of CAG repeats (Brinkman et al., 1997; Rosenblatt et al., 2006, 2012; Tabrizi et al., 2013; Langbehn et al., 2019).

### Epidemiology

Huntington's disease is a genetic disease, but there are sporadic or *de novo* cases that are now genetically proven to represent at least 5–8% of diagnosed patients (Almqvist et al., 2001; Ramos-Arroyo et al., 2005). A meta-analysis estimated the service-based worldwide prevalence of 2.7 cases per 100,000 and revealed an incidence of 3.8 per million per year (Pringsheim et al., 2012). Bates et al. (2015) reviewed the prevalence studies incorporating both genetic and clinical diagnostic standards which showed a greater prevalence and incidence in Western populations compared to the average population.

### Impaired Cognitive Domains

Evidence indicates that cognitive impairment in HD can precede motor symptoms of chorea by decades (Paulsen et al., 2008; Erkkinen et al., 2018), proving that cognitive impairments emerge both in the premanifest stage and manifest stage. The profile of cognitive decline in HD bears similarities to other disorders associated with subcortical dementia, and the cognitive dysfunction is characterized by psychomotor slowing a difficulty in the executive function, impairment in working, visual and verbal memory impairment, visuospatial dysfunction, sensory-perceptual dysfunctions, and impaired concentration or attention both in premanifest (Solomon et al., 2007; Tabrizi et al., 2009; Stout et al., 2011; Harrington et al., 2012; Unschuld et al., 2013) and manifest stage (Tabrizi et al., 2009; Stout et al., 2011, 2012; Unschuld et al., 2013; Mörkl et al., 2016), while cortically mediated processes, such as language and praxis are relatively spared (Erkkinen et al., 2018).

#### Psychomotor Slowing

The earliest change and the best predictor of HD progression was psychomotor slowing (Snowden, 2017) or the damage of information processing speed and motor skills (Paulsen, 2011). Patients with HD had difficulties in performing tasks that require simultaneous motor and cognitive processing, such as motor-cognitive dual-tasks (Fritz et al., 2016) or digit symbol subtest from WAIS-R (Bamford et al., 1989); Moreover, impairment of Symbol Digit Modalities Test (SDMT, participants use a key presented at the top of the test page to match symbols with numbers presented in horizontal rows. The task requires that the participant fills in the appropriate symbols below the matching numbers as quickly as possible.), Stroop Word Reading (a subset of the Stroop color word test mentioned above), and Paced Tapping Test [Participants tap on the left and right mouse buttons, alternating between thumbs, at 1.8 Hz (slow condition) or 3.0 Hz (fast condition). They first tap on the mouse buttons following a tone at the desired tapping rate; then the participants attempt to continue tapping at the same rate when the repetition of the tone is discontinued.] were observed in the patients with early HD after 24 months follow-up (Stout et al., 2012; Tabrizi et al., 2012). Actually, research showed that the patients with HD were mostly slowed in their cognitive processes rather than their actual hand movements (Aron et al., 2003).

The psychomotor slowing was also observed in the premanifest stage of patients with HD (Tabrizi et al., 2012, 2013). This is proved in most cross-sectional studies and longitudinal PREDICT-HD studies (Paulsen et al., 2006, 2008; Harrington et al., 2012; Dumas et al., 2013). Researchers administered the Maximum Tapping Speed Test (Participants tap at their maximum speed for 10 s using the non-dominant hand. Five trials are administered and the measure is the mean of the inter-tap intervals.), Stroop Test, Two Choice Reaction Time Task (One of the two adjacent circles on a touch screen turns green and the participants immediately press the circle. The measure is the mean movement time, which reflects the response selection processes.) and cued movement sequencing task (Filled circles are displayed in 12 vertical pairs along the bottom of a touch screen. Participants press sequentially the illuminated circles. The next circle is illuminated when the finger is lifted from the previous circle. Eight error free trials are administered.) to prodromal HD participants for the test of motor speed, showing a significant decline prior to years from the estimated diagnosis (Harrington et al., 2012).

#### Executive Dysfunction

Executive dysfunction was also noted in the disease duration both at the premanifest stage and at the manifest stage (Dumas et al., 2013; Mörkl et al., 2016). During the premanifest stage, impairment of the Stroop Test, Verbal Fluency Test, and TMT demonstrated executive dysfunction (Paulsen et al., 2006; Harrington et al., 2012). Still, some negative results from the premanifest stage were reported, partly due to the small sample sizes (Pirogovsky et al., 2007; van Walsem et al., 2010). A longitudinal study also confirmed the executive dysfunction at the premanifest stage (Lemiere et al., 2002; Solomon et al., 2008; Rupp et al., 2010). While in the manifest stage, the situation was different between the early and the late stage of HD.

In the comparison between the early and the late stage of patients with HD, the performance in the Number of Solved Problems [a part of the Tower of London Test, where the participants are given two boards (start board and goal board), where each board contains three pegs of descending lengths and three balls: one red, one yellow, and one blue ball. The participants are instructed to transform the start board ball arrangement to look exactly like the goal board ball arrangement using the smallest number of single-ball movements possible.] seemed to get worse with the progress of the disease (Mörkl et al., 2016). In the late stage, the everyday activities of patients with HD were deeply influenced by executive dysfunction, as the apathy/executive score accounted for significantly more unique variance in the instrumental HD Activities of Daily Living scale scores than did the motor score (Hamilton et al., 2003). Some other tests include the Wisconsin Card Sorting Test (WCST, participants are presented with the same four cards at the top of the screen on every trial and are asked to select which of these cards they would like to sort as the target card. The consistent presentation of the top cards allows the individuals to sort the target card on the basis of the color, shape, or number of stimuli. Subjects are told that the correct answer depends on a rule, but that they would not be told what the rule was. If the subjects sort the card into the correct deck, they receive “CORRECT” feedback. Once the subjects have made 10 consecutive correct selections, the rule changes so that another feature becomes the correct matching criteria.) (Paulsen et al., 1995), Clinical Rating Scales of Executive Dyscontrol (Duff et al., 2010) revealed an executive dysfunction also in patients with manifest HD as well.

#### Memory

The ability of the patients with HD in learning and retrieval of new information was impaired but, in contrast to AD, rapid forgetting was not so pronounced, and language ability was relatively preserved (Solomon et al., 2007). Gene carriers of patients with HD (preHD) s showed a deficit in learning and memory, including working memory, reflected by Letter-Number Sequencing, Dual Verbal Working Memory Test (Paulsen et al., 2006; Harrington et al., 2012) (participants name the color of digits presented serially every 1,500 ms, and then recall the serial order of the digits) and 2-back Working Memory (Harrington et al., 2012), and verbal memory tested through Phonemic Verbal Fluency (Harrington et al., 2012) and Hopkins Verbal Learning Test (HVLT, The task requires the participants to recall 12 words after examiner presentation over three trials. The raw score is the sum of the words recalled over the three trials. A second delayed recall score is the number of words recalled 20 to 25 min after the initial task) (Paulsen et al., 2008; Harrington et al., 2012). Interestingly, preHD research showed that lower HVLT-R (revised) scores were associated with closer proximity to the clinical diagnosis and smaller striatal volumes, reflecting that verbal episodic memory was affected in early preHD and may be impaired as striatal volumes decreased (Solomon et al., 2007). Nonetheless, memory decline at the premanifest stage was a slow process supported by a 12-month follow-up study (Tabrizi et al., 2011). Also in the manifest stage, both early and late stages of HD, cross-sectional study (Lawrence et al., 2000; Brandt et al., 2005; Tabrizi et al., 2009; Schneider et al., 2010) and longitudinal study (Bachoud-Lévi et al., 2001; Lemiere et al., 2002; Ho et al., 2003; Stout et al., 2012) showed impaired memory, including visual working or visuospatial recognition memory, working memory, and motor sequence learning. For example, the TRACK-HD study with 24 months follow-up showed that patients with early HD had worse performance in spotting the change task, elucidating visual working memory deficit (Stout et al., 2012).

#### Sensory-Perceptual Ability

One PREDICT-HD study showed that sensory-perceptual processing was the strongest unique predictor of time to diagnosis, after adjusting for the CAG-age product and Unified Huntington's Disease Rating Scale (UHDRS) motor score (Harrington et al., 2012). The sensory-perceptual ability included paced timing and emotion recognition.

##### Paced Timing

One important test for the ability of time recognition was Paced Tapping Test (Harrington et al., 2012). Many studies demonstrated impairments in the perception of time and the production of timed output in patients with preHD and HD (Hinton et al., 2007; Rowe et al., 2010; Harrington et al., 2012; Stout et al., 2012). One study reported that the timing task can be repeated longitudinally and the rate of decline increased with the estimated proximity to diagnosis, suggesting that timing may be a screening tool and outcome measure in preHD clinical trials to gauge therapeutically mediated improvement or maintenance of function (Rowe et al., 2010).

##### Emotion Recognition

One of the earliest cognitive impairments detected in preHD and early HD was the less accurate emotion identification (Johnson et al., 2007). Those with deficits of emotion recognition did remember what each emotion type meant, but they cannot recognize it upon presentation, especially the negative emotions (Tabrizi et al., 2009). The problems with disgust emotion recognition were most evident, followed by fear (Dumas et al., 2013).

PreHD showed a borderline overall impairment in recognizing the facial expressions of emotion, but statistical significance was only observed in disgust emotion (Gray et al., 1997). In the Benton Facial Recognition Test, participants are shown arrays of black and white faces and asked to match a sample face to either one out of six sample faces, or three out of six sample faces shown in different lighting and orientation conditions. Each correctly identified face earns one point. Poor performance of this test has indicated that patients with preHD had impaired emotion recognition (Paulsen et al., 2006). A series of TRACK-HD studies also showed that the correct number in the Negative Emotion Recognition Test (participants were asked to indicate the emotion expressed in each photograph by selecting from the words fear, disgust, happy, sad, surprise, angry and neutral) declined both in patients with preHD and early HD (Tabrizi et al., 2009, 2013; Stout et al., 2012); moreover, a significant difference between the patients with preHD and early HD was observed as well (Tabrizi et al., 2009, 2012), meaning that the emotion recognition ability got worse with the disease progression.

## Fragile X-Associated Tremor/Ataxia Syndrome

### Introduction

Fragile X-associated tremor/ataxia syndrome (FXTAS) is a progressive neurodegenerative disorder presenting with intention tremor, cerebellar ataxia, parkinsonism, and cognitive decline (Seritan et al., 2008; Apartis et al., 2012); peripheral neuropathy is also present commonly and typically develops before tremor and ataxia (Hagerman et al., 2007; Soontarapornchai et al., 2008; Apartis et al., 2012). Typical pathological hallmarks of FXTAS are the presence of eosinophilic ubiquitin-positive intranuclear inclusions in the neurons and astrocytes throughout the CNS of the affected individuals (Greco et al., 2002; Hagerman, 2013). There are a growing number of studies supporting the cognitive deficits in patients with FXTAS following a fronto-subcortical pattern.

### Pathogenic Gene

The pathogenic mutation of FXTAS was first described in a case report (Hagerman et al., 2001), with premutation repeat expansions (55–200 CGG repeats) in the 5'UTR of fragile X mental retardation 1 (FMR1) gene, which leads to increased levels of its mRNA and greater RNA gain-of-function toxicity (Tassone et al., 2000; Jacquemont et al., 2003; Hagerman and Hagerman, 2013). Generally, the average age of onset of this disease is 60.6 years, and the median survival is 21 years (Leehey et al., 2007). Interestingly, the greater CGG repeats size is associated with the earlier age of onset and perhaps earlier death (Leehey et al., 2008; Seritan et al., 2016).

### Epidemiology

Recent meta-analysis had determined that the prevalence of premutation CGG repeat expansions among the general population is ~1 in 150–300 for women and ~1 in 400–850 for men (Seltzer et al., 2012; Tassone et al., 2012; Hunter et al., 2014). However, the prevalence varied globally and was the highest in Israel, ~1 in 100 women (Toledano-Alhadef et al., 2001), and the lowest in Taiwan area ~1 in 1,674 women (Tzeng et al., 2005). Despite having a high prevalence, a large part of individuals with the FMR1 premutation did not have intellectual disabilities (Hagerman and Hagerman, 2013). Approximately 40% of men and 16% of women who carry premutation expansions of the FMR1 gene were affected (Jacquemont et al., 2004; Rodriguez-Revenga et al., 2009).

### Impaired Cognitive Domains

The cognitive decline affected 50% of men with FXTAS, and could be the initial symptom without obvious motor deficits; some may progress to frank dementia, while females were less affected, demonstrating the sex difference of the cognitive profile with FXTAS (Seritan et al., 2008; Juncos et al., 2011; Bourgeois, 2016). Moreover, the cognitive impairment was correlated with the CGG repeat number. Carriers with mid-range CGG repeats had the highest relative risk compared to the carriers with lower or higher CGG repeats (Sévin et al., 2009; Klusek et al., 2020).

The cognitive domains mainly affected in FXTAS were attention (Cornish et al., 2008), visuospatial processing (Hocking et al., 2012), executive function, and memory (Brega et al., 2008; Grigsby et al., 2008; Cornish et al., 2009).

#### General Intelligence

As for global cognitive performance, some reports have shown that FXTAS patients have a worse performance in the verbal IQ and performance IQ scores in MMSE (Schneider et al., 2012; Yang et al., 2013a) and Wechsler Adult Intelligence Scale–III (WAIS-III) compared to controls (Grigsby et al., 2008), reflecting a relative deficit in the global cognitive function, especially in male patients. But Birch et al. (2014) summarized the MMSE scores among FXTAS cases reported before 2014; the mean scores remained above the clinical cut-off point for cognitive dysfunction.

#### Attention

Many young patients with attention problems were diagnosed as having attention deficit and hyperactivity disorder (ADHD) before the onset of FXTAS (Farzin et al., 2006; Hunter et al., 2012). The deficit in visual attention detected by the WAIS–III Block Design Subtest showed a significant difference in male patients with FXTAS compared to normal controls, while asymptomatic premutation carriers do not show any difference (Grigsby et al., 2008). Another study on male premutation carriers showed a decline in performance for selective attention as age increased compared to controls, tested by the Everyday Attention-Map Search Task, a timed visual search task requires the participants to identify target symbols (e.g., a knife and fork sign representing restaurant facilities) from competing and irrelevant distracters on a large color map (Cornish et al., 2008). However, one study with mixed male and female patients showed no difference between patients with FXTAS and normal controls, neither did premutation carriers (Schneider et al., 2012), emphasizing sex difference in attention in both patients and premutation carriers.

#### Memory

Some research has found that both patients with FXTAS and premutation carriers without FXTAS symptoms had problems in memory. The memory domains affected mostly in FXTAS were verbal learning and memory together with working memory. One interesting finding was male patients or premutation carriers often showed memory problems, while women did not show any memory problems.

In detail, one study on male premutation carriers revealed significant impairments in learning and memory (Moore et al., 2004). Moreover, declarative verbal learning and memory are reflected by the Logical Memory Test (LMT, participants recall the details of a short story read aloud by the examiner and the total number of story elements recalled immediately after the story was read and after a 30-min delay are measured) and postinterference trial of the AVLT also impaired in FXTAS male patients (Grigsby et al., 2008). As for working memory, those male patients with FXTAS performed significantly worse on the WAIS–III Working Memory Index (Grigsby et al., 2008) and WAIS-III Letter-Number Sequencing (Brega et al., 2008) compared to the controls with normal FMR1 alleles. This situation was similar in male premutation carriers. When compared to the controls, they demonstrated an age-related decline in the performance on measures tapping the “central executive,” a crucial component of working memory (Cornish et al., 2009). However, in some studies with female-only or male-mixed cohorts, the decline in memory is not obvious. This was reflected by the California Verbal Learning Test (CVLT), a serial learning test, where List A with 16 words is presented five times in succession, in any order, after that, the second list B, unrelated to the first list, is read to the subjects; the subjects are then asked to recall List A, and after a 20-min interval, are asked again to recall List A (Yehuda et al., 1995) (Olichney et al., 2010; Schneider et al., 2012; Yang et al., 2013a). It was also evident in mutation carriers reflected by WAIS-III and Wechsler Memory Scale (WMS-III) (Schneider et al., 2012; Yang et al., 2013b).

#### Executive Function

Research has shown that performance in executive function was impaired both in male and female patients. The male patients with FXTAS got worse performance in the behavioral self-regulation than the controls, reflected by the lower scores in the Behavioral Dyscontrol Scale [BDS, a systematic tool assessing the different aspects of executive function, it includes nine items, such as “Rapidly tap twice with the dominant hand and once with non-dominant,” “Learning of a hand sequence (fist–edge–palm),” and so on], Stroop Test, and the test for verbal fluency, such as COWAT Total Score (Brega et al., 2008). The performance of BDS also declined in another study including older women (Schneider et al., 2012). A study by Yang et al. (2013a) showed similar worse performance with BDS, COWAT, and Stroop Test tested in a cohort with male and female patients.

The manifestations of cognitive impairment among the asymptomatic male and female carriers are less well understood but have come under increased scrutiny (Grigsby et al., 2014). According to the few instances of published data, the researchers found that male premutation carriers also had significant impairments on the tests of executive function compared to controls (Moore et al., 2004). Female patient carriers showed relatively lower scores in BDS than the normal control (Yang et al., 2013b).

#### Information Processing Speed

Speed of information processing was also compared both in patients with FXTAS and premutation carriers and also showed sex differences. In the male group, patients with FXTAS presented a significantly worse performance in information processing speed than the controls. They had a deficit in SDMT and the processing speed index of WAIS-III (Grigsby et al., 2007). The Symbol Search Subtest and SCWT were observed (Brega et al., 2008; Grigsby et al., 2008) and when male and female participants were combined, a difference was also observed in WAIS-III (Schneider et al., 2012; Yang et al., 2013a) and the oral version of the SDMT (O'Keefe et al., 2018). However, some controversial conclusions have been drawn in different studies in the group containing female patients. One study found that female patients and premutation carriers showed borderline significance compared to the normal controls (Yang et al., 2013b). While in a recent study, researchers examined language processing skills in 46 women with the premutation and 56 controls. They found a pattern of inefficient language processing (Nayar et al., 2019). Therefore, the cognitive domain in information processing speed requires further detailed study.

## Spinocerebellar Ataxias

### Introduction

Spinocerebellar ataxia (SCA) is a type of autosomal dominantly inherited disease characterized by a progressive loss of balance and coordination accompanied by slurred speech (Klockgether et al., 2019). More than 40 SCA subtypes were discovered so far (Bhandari et al., 2020), and 12 of them are caused by expanded polynucleotide repeats in the coding exons or the non-coding area (Klockgether et al., 2019). The representative pathology change of SCAs is summarized in one excellent review (Seidel et al., 2012); briefly, neuropathological data for the SCAs showed considerable overlap in the neurodegenerative pattern in end stage patients, revealing damage to the cerebellum and/or its neuronal connections, sometimes to the cerebral cortex (Table 1).

**Table 1:** The pathogenic mutation, impaired cognitive domains, and atrophy patterns of SCAs with cognitive dysfunction.

**SCAs**	**Pathogenic mutation Normal repeat number Pathogenic repeat number**	**Impaired cognitive domains**	**MRI or atrophy pattern**
**Mutation in coding exons**			
SCA1	(CAG)n in Ataxin-1 6–39 40–82	Executive dysfunction Attention Psychomotor speed	Brainstem and cerebellar atrophy
SCA2	(CAG)n in Ataxin-2 15–24 32–200	Executive dysfunction Attention Psychomotor speed	Brainstem and cerebellar atrophy
SCA3	(CAG)n in Ataxin-3 13–36 61–84	Executive dysfunction Visuospatial function Attention Psychomotor speed	Brainstem and cerebellar atrophy
SCA6	(CAG)n in CACNA1A 4–18 21–29	Executive dysfunction Visuospatial function Attention (±) Psychomotor speed (±)	Cerebellum
SCA7	(CAG)n in Ataxin-7 4–35 37–306	Executive dysfunction Recognition memory Psychomotor speed	Brainstem and cerebellar atrophy
SCA17	(CAG)n in TBP 25–42 47–63	**Dementia** Executive dysfunction anterograde memory Visuospatial function Attention Psychomotor speed	Cerebellum and cerebral cortex, brainstem (late stage)
DRPLA	(CAG)n in Atrophin 1 7–34 49–88	**Dementia** Memory impairment Visuospatial function Attention	Brainstem and cerebellar atrophy, cerebral white matter, thalamus
**Mutation in non-coding area**			
SCA8	(CTG)n in Ataxin-8(KLHL-1) 3'UTR 16–34 >74	Executive dysfunction Immediate recall and delayed recall Visuospatial function Attention Psychomotor speed	Cerebellum
SCA10	(ATTCT)n in Ataxin10 intron 10–20 500(550)−4500	Executive dysfunction Memory impairment Visuospatial function	Cerebellum
SCA12	(CAG)n in PPP2R2B 5'UTR 7–45 55–78	**Dementia** Anterograde memory Hemi-inattention (one case)	Cerebellum and cerebral cortex, brainstem (late stage)

*CACNA1A, Calcium Channel, Voltage-Dependent, P/Q Type, Alpha-1A Subunit; KLHL-1, Kelch-like protein 1; PPP2R2B, Protein Phosphatase 2, Regulatory Subunit B, Beta; TBP, TATA Boxing-Binding Protein*.

### Pathogenic Genes

According to the literature published to date, cognitive dysfunction in polyglutamine ataxias is prominent in SCA1, SCA2, SCA3, SCA6, SCA7, SCA17, and dentatorubral-pallidoluysian atrophy (DRPLA), which are caused by expanded CAG repeats in the coding exons (Schöls et al., 2004; Gatchel and Zoghbi, 2005; Lindsay and Storey, 2017; Giocondo and Curcio, 2018). As for mutation in the non-coding area, patients with SCA8, SCA10, and SCA12 present cognitive dysfunction or dementia (Gatchel and Zoghbi, 2005; Lindsay and Storey, 2017; Giocondo and Curcio, 2018), (CTG)n repeats in Ataxin8 3'UTR causes SCA8 (Koob et al., 1999), (ATTCT)n in Ataxin10 intron results in SCA10 (Matsuura et al., 2000), and (CAG)n in PPP2R2B 5'UTR leads to SCA12 (Holmes et al., 1999). The pathogenic mutations and repeat numbers of each SCA with cognitive impairment are listed in Table 1.

### Epidemiology

The prevalence of SCAs assessed in population-based studies ranged from 0 to 5.6 cases per 1,00,000 individuals, with an average of 2.7 cases per 1,00,000 individuals (Ruano et al., 2014). But the relative frequency of the different SCA subtypes showed marked geographical and ethnic variability (Sequeiros et al., 2012).

Generally, SCA1, SCA2, SCA6, SCA7, and SCA8 had a prevalence of over 2%, and the remaining subtypes were thought to be rare (prevalence < 1%) (Schöls et al., 2004). SCA3/MJD was the most common SCA worldwide (20–50% of families with SCA), followed by SCA2 and SCA6 (Hersheson et al., 2012).

### Impaired Cognitive Domains

Although intellectual symptoms were not part of the typical clinical spectrum of SCAs, neuropsychological testing revealed subtle abnormalities in general intelligence, executive dysfunction, memory, visuospatial function, attention, and psychomotor speed (Giocondo and Curcio, 2018).

#### General Intelligence

Generally, the severity of global cognitive function differed in the subtypes of SCAs. The MMSE scores of patients with SCA1, SCA3, and SCA7 were significantly lower than the controls, though not they did not reach the line of 24 (Bürk et al., 2001; Chirino et al., 2018; Tamura et al., 2018). The scores of patients with SCA2, SCA6, SCA8, and SCA10 were close to controls (Lilja et al., 2005; Fancellu et al., 2013; Moro and Teive, 2016; Tamura et al., 2017), which proved that despite general cognitive dysfunction being common in SCA1, SCA3, and SCA7, patients did not always meet the diagnosis criteria of dementia. Furthermore, patients with SCA12, SCA17, and DRPLA got a much lower score, reaching the borderline of 24 in the late stage of these diseases (O'Hearn et al., 2001; Nielsen et al., 2012; Sugiyama et al., 2018), indicating the presence of dementia in these subtypes.

#### Executive Function

First of all, executive dysfunction was observed in most of the SCAs described above. In detail, it was the most commonly reported cognitive feature in patients with SCA1, SCA2, and SCA3 (Bürk et al., 2001, 2003; Klinke et al., 2010; Ma et al., 2014), and patients with SCA1 were more prominent and with higher error rates than the patients with SCA2 and SCA3 (Bürk et al., 2003). The results in SCA6 were controversial. One systematic study in SCA6 revealed no significance between the patients and the controls (Globas et al., 2003). But some articles indicated that patients with SCA6 showed deficits on the Hayling Sentence Completion Task (Garrard et al., 2008). As for SCA7, selective deficits with a cognitive profile similar to SCA 1 and SCA 6 were reported, primarily in the executive function with impairment in phonemic fluency, the Hayling Test, and in the response inhibition on the Stroop Test (Sokolovsky et al., 2010). One comprehensive evaluation on the cognition of patients with SCA8 showed impairment in the executive dysfunction with WAIS-R Picture Completion and Arrangement (Lilja et al., 2005). Another part of executive dysfunction, verbal fluency, and naming, also showed a decline in the patients with SCA8 (Lilja et al., 2005). The SCA10 also got worse performance in CDT and verbal fluency compared to normal controls (Moro et al., 2017). As for SCA12, the evidence was rare for executive dysfunction. But dementia in SCA17 was common, and executive control deficit and impaired problem-solving skills were reported recently in an Irish family; a relatively lower score of WCST also reflected in the impairment of executive function (Olszewska et al., 2019), and the pattern mimics FTD partly with a prominent deficit in verbal fluency (Bruni et al., 2004). Furthermore, dementia was reported in DRPLA but details of cognitive decline are relatively sparse.

#### Memory

In SCA1, there were some controversial conclusions about verbal memory impairment. Some studies found that verbal memory was impaired prominently in SCA1, which was more severe than in patients with SCA2 and SCA3, reflected by the immediate recall of the Randomized Categories List (Four words of four different categories were presented in a randomized order) (Bürk et al., 2003), while another study using the word recall from the Alzheimer's Disease Assessment Scale-cognitive (ADAS-Cog) showed that immediate and delayed recall were relatively reserved in SCA1, but impaired in SCA2 and SCA3 (Ma et al., 2014). In SCA6, the impairment in the working memory, verbal, or visuospatial memory was not significant (Globas et al., 2003). While patients with SCA7 showed recognition memory deficits both at the baseline and follow-up, reflected by the Recognition Memory Test (words/face), though the sample size of the study is relatively small (Moriarty et al., 2016). Also, immediate recall and delayed recall were impaired in patients with SCA8 (Lilja et al., 2005). The clinical data studying the memory problem of SCA10 is relatively rare, with one patient reporting mild memory deficit (Teive et al., 2004) and one patient showing a learning problem (Raskin et al., 2007). Poor anterograde memory formation was observed in 2 out of 10 patients with SCA12 in the late disease stage (O'Hearn et al., 2001), and memory impairment was noted in the Indian SCA12 family, though the tests used were unclear (Fujigasaki et al., 2001). Anterograde episodic memory and working memory were clearly affected in one patient with SCA17, together with immediate and delayed recall reflected by RCFT (Olszewska et al., 2019). Memory disturbance was observed in patients with DRPLA as well (Muñoz et al., 2004). Reduced visual memory was observed and measured on an unnamed task requiring memory for complex block constructions in an adult patient (Vinton et al., 2005).

#### Visuospatial Function

Visuospatial function, as tested with the RCFT with respect to copying, delayed recall, and proportional recall of the initial copy, was unimpaired in SCA1, SCA2, and SCA3 types (Bürk et al., 2003). While another study showed that the patients with SCA3 got worse performance in the visuospatial and constructional tasks, such as Block Design with and without a time limit (Kawai et al., 2004). The patients with SCA6 showed a deficit in figure copy, a part of RCFT and TMT reflecting visuospatial executive function (Rentiya et al., 2018). In 7 Zambian SCA7 families, 15 out of 19 patients showed decreased visual acuity (Atadzhanov et al., 2017). But research focusing on visuospatial function in SCA7 was rare. The situation was similar in SCA12; Visuoperceptual and constructive functions of patients with SCA8 were preserved (Lilja et al., 2005). Patients with SCA10 performed worse than the controls in CDT, revealing that the patients got impairment in the visuospatial function (Moro et al., 2017). A case with severe and rapidly progressing cognitive phenotype in a SCA17-family showed visuospatial impairment presenting an inability to copy simple drawings or assemble simple block designs. However, the patient had only marginally expanded CAG/CAA repeats in the TBP gene (Nielsen et al., 2012). A special case was reported about three generations of men in an Australian Macedonian family with DRPLA, the adult-onset patients showed a reduced visual memory for complex block constructions, and the elderly onset grandfather was presented with inaccuracy in the copying of the RCFT (Vinton et al., 2005).

#### Attention

Patients with SCA1 showed attention impairment reflected by Digit Span Forward, while the patients with SCA2 and SCA3 showed no deficit in it, but the SCA2 sample size was relatively small with only two patients (Ma et al., 2014). Therefore, another study with 22 patients with SCA2 showed a significant difference compared to the controls, with the performance getting worse after a 2-year follow-up (Fancellu et al., 2013). Moreover, one study with 15 patients with SCA3 also concluded that patients with SCA3 performed worse than the normal controls in the attention reflected by the Symbol Counting Test (a subtest of the CI Test) (Klinke et al., 2010). This indicates that attention was impaired in SCA1, SCA2, and SCA3, particularly in SCA2 (Moriarty et al., 2016). In SCA6, debate still exists; some studies have shown no significance in terms of attention (Globas et al., 2003; Garrard et al., 2008; Tamura et al., 2017). Klinke et al. (2010) reported that patients with SCA6 were significant, although mildly, impaired in attention with a deficit in Symbol Counting Test. While a small-sample-size study showed that the patients with SCA7 presented no attention problem (Sokolovsky et al., 2010), in SCA8, the deficit in attention and information process were observed and reflected by the part of WAIS-R, Digit Span, and Digit Symbol (Lilja et al., 2005). Details about attention in SCA10 and SCA12 were rare. Hemi-inattention was only reported in 2 out of 10 patients with SCA12 (O'Hearn et al., 2001). The patients with SCA17 were dominated by profound and disproportionate attention (Olszewska et al., 2019). Bruni et al. (2004) showed us that more than half of the 16 affected SCA17 patients showed attention or planning deficit, but the questionnaires used were unclear. The patients with DRPLA also showed deficits in attention, and one of the neuropsychological features of juvenile-onset DRPLA was attention-deficit-hyperactivity disorder (Licht and Lynch, 2002).

#### Psychomotor Speed

Studies associated with psychomotor speed were relatively scarce in SCAs. A longitudinal investigation of SCA1, SCA2, SCA3, SCA6, and SCA7 cognition progression showed impairment in Stroop C-W Test and oral SDMT, which reflected a relatively slow psychomotor speed of all the SCA subtypes, but the sample sizes were small in all the subtypes (Moriarty et al., 2016). Moreover, another study with eight SCA1, two SCA2, and eight SCA3, also showed deficits in Stroop Test (Ma et al., 2014).

As for SCA6, the cognitive investigation of the patients with the Stroop Test showed no significance compared to the controls (Rentiya et al., 2018), while another study using the Symbol Counting Test to evaluate the processing speed found the difference between SCA6 and the controls (Klinke et al., 2010). The study by Sokolovsky et al. (2010) showed that two out of three patients with SCA7, who were administered the SCWT presented a declined psychomotor speed. The situation was similar in SCA8, the researchers used the CogniSpeed software to examine the speed and accuracy of information processing and attention functioning; the different reaction times of different objects showed the impairment in the psychomotor speed of the patients with SCA8 (Lilja et al., 2005). Psychomotor speed substantially decreased in the patients with SCA17 as well (Olszewska et al., 2019). However, data associated with SCA10, SCA12, and DRPLA are rare, meaning the impairment of psychomotor speed was unclear in these three subtypes.

## Other REDs With Cognitive Dysfunction

With the recent advances in sequencing technology, researchers have been able to discover the pathogenic mutation of RED with a much faster speed. Part of these diseases showed cognitive dysfunction or mild cognitive impairment, but articles focusing on the cognitive domains are relatively rare. Therefore, this review presents these diseases in Table 2 and introduces them briefly.

**Table 2:** The pathogenic gene, repeat numbers, and impaired cognitive domains in other repeat expansion diseases (REDs) with relatively rare data.

**Disease**	**Gene (protein)/** **repeat unit/position**	**Normal/** **pathogenic repeat number**	**Impaired** **cognitive** **domains**
Myotonic Dystrophy 1 (MD1)	DMPK (CTG)n 3'UTR	5–37 >50	Attention deficit/hyperactivity disorder and anxiety disorder
Progressive Myoclonus Epilepsy of Unverricht-Lundborg type (EPM1)	CSTB (cystatin B) (CCCCG CCCCGCG)n 5'UTR	2–3 >30	Mild decline in intellectual performance over time adult patients: deficits in executive function, information processing speed, visuospatial ability, working memory and perceptual reasoning
Friedreich ataxia (FA/FRDA)	FXN (frataxin) (GGA)n intron 1	6–27 44–1,700 (600–900 GAA repeats being the most common)	Visuoconstructive abilities, verbal fluency, attention, information processing speed and planning, and implicit learning
Huntington Disease like 2 (HDL2)	JPH3 (junctophilin 3) (CAG/CTG)n a variably spliced exon (exon 2A)	7–28 66–78	Severe decline in psychomotor speed and dexterity, together with visuo-constructive ability
Cerebellar ataxia, neuropathy, vestibular areflexia syndrome (CANVAS)	RFC1 (replication factor complex subunit 1) (AAGGG)n intron 2	11 (<15) Over several thousand	Mild cognitive impairment or mentally slow, executive dysfunction, short-term memory deficit

### Myotonic Dystrophy Type 1

Myotonic dystrophy type 1 (DM1), also called Steinert disease, is a progressive neuromuscular disease caused by a genetic mutation with autosomal dominant transmission and characterized by a wide variation in the neuromuscular symptoms and multisystem involvement. Generally, DM1 can be categorized into five phenotypes: congenital, infantile-onset, juvenile-onset, adult-onset, or late-onset with distinct clinical features for each phenotype (De Antonio et al., 2016). Cognitive dysfunction can be observed in the adult-onset subtype, and intellectual disability is more often observed in patients with congenital and infantile-onset (Udd and Krahe, 2012). The pathogenic gene of DM1 was discovered in 1992, with (CTG)n expansions in 3'UTR of DMPK gene ranging from 51 repeats to several thousand, whereas the healthy controls carry 5–37 repeats (Breton et al., 2020).

Although DM1 is defined as a muscular disease, altered cognitive functioning is an important feature of DM1, especially the infantile-onset and adult-onset subtypes (Meola and Sansone, 2007). The infantile-onset patients always present with intellectual disability, and the individuals with adult-onset can experience cognitive difficulties that may be linked to frontal lobe function (Langbehn et al., 2021). In detail, the adult-onset patients often showed deficits in executive function, information processing speed in complex executive functioning tasks, visuospatial ability, working memory, and perceptual reasoning (Gallais et al., 2017; Fujino et al., 2018; Langbehn et al., 2021). Interestingly, the dysfunction in perceptual reasoning and processing speed was associated with muscle function, and the researchers found an inverse association between the hippocampal volume and in both processing speed and perceptual reasoning in the DM1 group (Langbehn et al., 2021). The cognitive state of patients with DM1 also plays an important role in quality of life (Fujino et al., 2018). As for infantile-onset patients, reduced IQ values, attention deficit, a deficit in visuospatial function, autism spectrum disorder, communication problems, and social anxiety were summarized in an excellent review (Gourdon and Meola, 2017). The problems with communication and fatigue appeared to have the greatest impact on children's life (Johnson et al., 2014). Importantly, the cognitive dysfunction symptoms could be the only manifestation in infantile-onset patients, so parents and doctors should pay more attention to the cognition change in children.

### Progressive Myoclonus Epilepsy of Unverricht-Lundborg Type

Progressive myoclonus epilepsy (PME) is a type of disease characterized by myoclonus, epilepsy, and progressive neurological deterioration. Unverricht-Lundborg disease (ULD), also called EPM1 is the most common type of PME; It is an autosomal recessive disease caused by the mutation of the cystatin B gene (CSTB) (Lehesjoki and Koskiniemi, 1998). Although some case reports presented traditional mutation of CSTB resulting in ULD, such as c132-134del mutation (Assenza et al., 2017) and point mutations in CSTB (Joensuu et al., 2008), the expanded dodecamer repeats mutation, (CCCCGCCCCGCG)n in the 5'UTR, accounts for ~90% of ULD throughout the world; moreover, about 99% of the affected Finnish cases are homozygotes for expanded alleles (Kälviäinen et al., 2008). The normal size of dodecamer repeats is two or three copies, while the patients usually present with an unstable expansion of at least 30 copies of the dodecamer repeat (Joensuu et al., 2008). Generally, the age of symptom onset in ULD is 6–15 years (Shahwan et al., 2005).

The overall IQ of clinically diagnosed patients with ULD has previously been reported to be diminished by 10 points over 10 years, reflected by the Wechsler Verbal or Terman-Merrill-Lehtovaara scale (Koskiniemi, 1974). This was confirmed in a Finnish nationwide study, demonstrating that an earlier age at onset for ULD and longer disease duration were associated with lower IQ performance (Hyppönen et al., 2015). Past studies have shown that cognitive impairment may be absent or vary from mild to moderate, but two studies demonstrated the cognitive profiles of ULD, revealing the impairment of abstract reasoning, short-term memory, attention, and executive function, especially the executive dysfunction (Ferlazzo et al., 2009; Giovagnoli et al., 2009). Moreover, the processing and execution were significantly associated with diagnosis, disease duration, and education (Giovagnoli et al., 2009). While another study demonstrated that 55% (11 out of 20 patients) of patients with ULD showed mild to moderate cognitive impairment, performing worse in short-term memory and executive function tasks, and the degree of impaired performance on some memory tests was associated with the duration of disease, while the performance in the executive function was related to the severity of myoclonus (Ferlazzo et al., 2009). One untypical sign of ULD was reported in 2008, showing rapidly progressive dementia in one patient; furthermore, three out of seven patients showed different degrees of intellectual dysfunction, disclosing verbal and performance IQ, and memory and reading ability impairment in patients with ULD, consistent with the description of mild cognitive dysfunction in patients with ULD (Chew et al., 2008).

### Friedreich Ataxia

Friedreich ataxia (FRDA) is the most common autosomal recessive ataxia. Clinically, Friedreich ataxia is characterized by an early-onset progressive gait and limb ataxia, dysarthria, loss of vibration, and proprioceptive sense, together with some complications, like cardiomyopathy and diabetes (Fogel and Perlman, 2007). In about 98% of patients, the disease is caused by a triplet GAA expansion within the first intron of the frataxin gene found on chromosome 9q13 (Campuzano et al., 1996). Generally, the unaffected individuals were presented with 6 to 27 repeats, while ~96% of FRDA patients have homozygous GAA repeat expansions ranging from 44 to 1,700 repeats, with 600–900 GAA repeats being the most common; moreover, large interruptions in the GAA repeats are very rare in patients with FRDA (Al-Mahdawi et al., 2018). The mean age of the onset of FRDA is 15 years, with most cases developing by age 25, although rare cases of late onset FRDA (26–39 years), or very late onset FRDA (40 years or over) have been reported (Al-Mahdawi et al., 2018). Interestingly, neuroimaging does not show progressive cerebellar degeneration commonly observed in the autosomal dominant hereditary ataxias. The pathological changes primarily affect the spinocerebellar tracts, posterior columns, and to a lesser extent the corticospinal tracts (Kawai et al., 2009).

Although the presence of epilepsy or cognitive or psychiatric symptoms is not typically seen in FRDA, there are still some studies addressing the cognitive profiles of FRDA. The performance of general intelligence in patients with FRDA was worse than controls, but most of them were within the normal range reflected by the WAIS (Mantovan et al., 2006). One study with genetically undiagnosed patients with FRDA showed that they had slowed information processing speed, as indicated by lengthened simple visual reaction time and increased color-word interference in the Stroop task (White et al., 2000). The first comprehensive study of cognitive function in the genetically confirmed FRDA demonstrated that patients with FRDA presented impairments of visuo-constructive abilities, verbal fluency, attention, information processing speed and planning, and implicit learning; furthermore, the researchers also reported that the GAA expansion size did not correlate with neuropsychological performance, but patients with longer disease duration showed worse performance in the Stroop Interference Task and the Tower of London (Mantovan et al., 2006). The cognitive domains such as working memory, emotional recognition, visual reasoning, and executive function were also impaired (Costabile et al., 2018; Sayah et al., 2018). One recent structural MRI study of patients with FRDA also confirmed a correlation between Lobule IX volume and the impaired visuospatial functions (Cocozza et al., 2020), and one resting-state functional MRI (RS-fMRI) analysis on patients with FRDA found an altered brain functional connectivity in patients (Cocozza et al., 2018), expanding knowledge about the physiopathology of cognitive impairment in FRDA.

### Huntington Disease-Like 2

Huntington disease-like 2 is a rare neurodegenerative disease with clinical manifestations that are similar to HD. The causative mutation is a CTG/CAG expansion in junctophilin3 (JPH3), which encodes JPH3 protein, and the expansion is located in an alternatively spliced exon, exon 2A (Holmes et al., 2001). The normal repeat number is 7–28, while the pathogenic number ranges from 66 to 78 (Gatchel and Zoghbi, 2005). Typically, HDL2 is presented in the midlife of the patients with a relentless progressive triad of movement, emotional, and cognitive abnormalities which lead to death from 10 to 20 years (Anderson et al., 1993), and the age at onset of the HDL2 shows a negative correlation to the repeat size (Anderson et al., 2017).

The HD clinical spectrum has a great clinical resemblance to HD; one recent MRI study compared the difference between HDL2 and HD, showing no significant difference with respect to mean age at MRI, disease duration, abnormal triplet repeat length, or age at disease onset, except the smaller thalamic volumes in the patients with HDL2 (Anderson et al., 2019). The general intelligence reflected by MMSE was impaired in the patients with HDL2 and the scores of most case reports were below 24, meeting the diagnostic criteria of dementia (Fischer et al., 2012; Vasconcellos et al., 2017). But its neurocognitive characterization is poorly researched systematically. While in 2020, Ferreira-Correia et al. (2020) summarized 7 pure reported HDL2 cases containing neuropsychological deficits in HDL2, showing a severe decline in psychomotor speed and dexterity, together with visuo-constructive ability; while the executive function, memory and learning, attention, and concentration were presented moderately in impaired functions. Furthermore, working memory tasks were generally within normal limits for the majority of patients.

### Cerebellar Ataxia, Neuropathy, Vestibular Areflexia Syndrome

Cerebellar ataxia, neuropathy, vestibular areflexia syndrome is a disease characterized by progressive unsteadiness, generally starting in the sixth decade and dry spasmodic cough, with sensory symptoms, oscillopsia, dysautonomia, and dysarthria (Cortese et al., 2020). Recently, the pathogenic gene, a biallelic intronic repeat expansion in the replication factor complex subunit 1 (RFC1), was identified, adding a new member to the list of REDs (Cortese et al., 2019). The pentanucleotide is generally repeated 11 times, often <15 repeats, while in the patients with CANVAS, the repeat numbers expanded from several hundred to over 2,000 (Gisatulin et al., 2020; Dominik et al., 2021). The mean onset for any neurological sign of CANVAS (cough excluded) is 52 years, albeit the onset might range between 19 and 76 years (Sullivan et al., 2021).

Little evidence was noted in the aspect of cognitive dysfunction in CANVAS, except for one article delineating the full phenotypic spectrum of this disease (Traschütz et al., 2021). The author noted that 25% (13/52) of patients showed cognitive impairment; in detail, most of them had mild cognitive impairment, and half of the 13 patients were presented with mentally slow, about two patients showed executive dysfunction, and 1 patient had a short-term memory deficit; however, the neuropsychological tests used were unclear, and the sample size was small; so further investigation is required to demonstrate the cognitive dysfunction in CANVAS.

## Diagnosis and Intervention

With the description above, we concluded that different REDs presented with different characteristics in cognitive domains. Moreover, some subtle alterations in cognition may help us with the early diagnosis of diseases or differential diagnosis from others. As for disease management, two important aspects were included: disease-specific therapies and non-specific intervention. With relatively limited progress in disease-specific drugs or treatment, two important non-specific interventions, including exercise and cognitive intervention, will be summarized.

### Auxiliary Diagnosis

In NIID, most patients may present dementia symptoms, while rare evidence or longitudinal study showed which cognitive domain was impaired first; therefore, this is still an important issue that requires further research. Furthermore, the pattern of cognitive impairment of NIID resembles other neurological diseases with white matter damage or other types of subcortical dementia; this is a vital differential point to differentiate cortical dementia, such as AD and FTD.

The FTD patient group was characterized by progressive deterioration in behavior, speech production, or language, with a relative sparing of memory and visuospatial function. While in the C9FTD group, the C9ORF72 mutation was associated more frequently with bvFTD, presented with inappropriate behavior and agitation, this is in contrast to patients with bvFTD who have a GRN mutation, in whom apathy dominates the clinical spectrum (Beck et al., 2008; Pickering-Brown et al., 2008).

In HD, the earliest cognitive impairments detected in preHD and early HD are psychomotor slowing and the less accurate emotion identification; moreover, psychomotor slowing is the best predictor of HD progression as well (Paulsen, 2011; Snowden, 2017), so the appearance of psychomotor slowing in preHD individuals may reveal the onset of HD symptom. As for the predicting value of diagnosis, the sensory-perceptual processing was the strongest unique predictor in the timely diagnosis (Harrington et al., 2012).

With regard to FXTAS, the original major diagnostic criteria for FXTAS comprise intention tremor, ataxia, and the middle cerebellar peduncle (MCP) sign. Minor diagnostic criteria include moderate memory and executive functional deficits as well (Hagerman and Hagerman, 2016). Moreover, the cognitive deficit could be the initial symptom without obvious motor deficits; so cognitive assessment could also do a favor for the diagnosis of FXTAS.

The impaired cognitive domains of SCAs were summarized in Table 1, and we concluded that SCA12, SCA17, and DRPLA could develop dementia with the disease progression. SCA6, SCA8, and SCA10 could be defined as mild dysexecutive syndromes and the impaired brain region was prominent in the cerebellum, while in the remaining SCA subtypes including SCA1, SCA2, SCA3, and SCA 7, more extensive deficits were noted. This conclusion was consistent with another review (Lindsay and Storey, 2017).

### Non-Specific Intervention

With the early diagnosis value of cognitive assessment, patients with these neurodegenerative diseases may benefit from timely intervention. Integrative approaches come into insight. Lifestyle management, cognitive intervention, and other non-invasive methods could be combined with therapeutic treatments.

Endurance exercise, as the most common physiotherapy, had well-established, powerful benefits to multiple organs and helped preserve memory and cognition, and improved symptoms of neurodegenerative diseases (Di Liegro et al., 2019). In HD, a study of 12 male patients with HD and 12 healthy age- and sex-matched controls completing 6 months of cycling exercise at 65% VO_2_ peak, the UHDRS and neuropsychological parameters did not decline, suggesting a generalized stabilization of disease progression (Frese et al., 2017). Most studies reported no related adverse events in response to training in the group of patients with HD (Mueller et al., 2019). The exercise was also one of the most effective non-pharmacological methods that promoted neurogenesis and were beneficial in premutation individuals having FXTAS. It can also be beneficial for psychiatric symptoms (Sodhi and Hagerman, 2021). In SCAs, the positive effects of exercise were noted in SCA2 (Velázquez-Pérez et al., 2019), SCA3 (de Oliveira et al., 2018), SCA6 (Ilg et al., 2009), and SCA7 (Tercero-Pérez et al., 2019) reflected by the improvement of motor or gait performance, postural, and coordinative deficits. However, the significance of physical exercise was unclear in NIID and C9FTD.

In recent years, the potential for developing cognitive or behavioral interventions has emerged as an important element to support neuroprotection, promote healthy cognitive aging, and preserve cognition in neurodegenerative diseases. In FTD, effort has been dedicated to developing the management of difficult or inappropriate behavior and language deficits, such as the Tailored Activities Program (TAP), which directly targets a specific behavior and redirects it into personalized activities (O'Connor et al., 2016). Five patients with SD, completing a 2-month, online word training program showed a clear improvement in language function, such as naming and in the comprehension of verbal instructions (Savage et al., 2014). While in the HD group, the limited study showed the therapeutic effect of cognitive intervention, except Emma's randomized feasibility study showing the barriers of cognitive intervention in HD (Yhnell et al., 2020). However, researchers still hold a positive view of the function of cognitive intervention (Papoutsi et al., 2014). Evidence about cognitive intervention effectiveness was lacking in patients with FXTAS (Sodhi and Hagerman, 2021). In SCAs, one randomized controlled trial (RCT) that evaluated tele-coaching on psychological adjustment over 3 months in 18 patients with SCA showed improved vitality, anxiety/depression, and locus of control.

## Conclusion

The present review summarizes the pathogenic gene, epidemiology, and cognitive domains affected in multiple REDs, such as NIID, SCAs, C9FTD, FXTAS, HD, and others (DM1, PME, FRDA, HDL2, and CANVAS), demonstrating a high prevalence of cognitive dysfunction in REDs, and provided a new aspect for differential diagnosis and the disease intervention of these diseases.

## Author Contributions

LS and BJ were involved in the review design, modification, and revision of the manuscript. SZ searched and reviewed the articles, and wrote the manuscript. All authors contributed to the article and approved the submitted version.

## Funding

This study was supported by the National Key R&D Program of China (No. 2020YFC2008500), the National Natural Science Foundation of China (Nos. 81671075, 81971029, 82071216, 81901171), and the Hunan Innovative Province Construction Project (No. 2019SK2335).

## Conflict of Interest

The authors declare that the research was conducted in the absence of any commercial or financial relationships that could be construed as a potential conflict of interest.

## Publisher's Note

All claims expressed in this article are solely those of the authors and do not necessarily represent those of their affiliated organizations, or those of the publisher, the editors and the reviewers. Any product that may be evaluated in this article, or claim that may be made by its manufacturer, is not guaranteed or endorsed by the publisher.
