# Supplementary material for: Cognitive Dysfunction in Repeat Expansion Diseases: A Review
Source: Front Aging Neurosci. 2022 Apr 11;14:841711. doi: 10.3389/fnagi.2022.841711 (PMC9036481; doi:10.3389/fnagi.2022.841711)
Supplement: Supplementary file 1 [file Table_1.DOCX]

Supplementary table 1: a brief summary of the neuropsychological tests used in these researches

| cognitive domain | Scales/neuropsychological tests |
| --- | --- |
| estimation of verbal and performance IQ | Wechsler Adult Intelligence Scale |
|  |  |
| assessment of dementia | Mini Mental State Examination (MMSE) |
|  | Montreal Cognitive Assessment (MoCA) |
|  |  |
| Episodic memory | The Rey Auditory Verbal Learning Test(RAVLT) |
|  | The recall component of the Rey-Osterrieth Complex Figure Test |
|  | The short story test |
|  |  |
| Verbal memory  (semantic memory) | Reproducing a prose passage from the WMS directly and 30 min after presentation. |
|  | Patients were asked for immediate and delayed recall of 16-item word lists. |
|  | Digit span(short-term verbal memory test) |
|  | short-term and long-term episodic verbal memory test(immediate and delayed recall of a short tale) |
|  | California Verbal Learning Test (CVLT) |
|  | the Hopkins Verbal Learning Test-Revised(HVLT)(immediate recall and delay recall) |
|  | Rey Auditory Verbal Learning Test (RAVLT) |
|  | Recognition Memory Test for words |
|  |  |
| Visual memory | Recognition Memory Test for faces |
|  | Spot the Change task（for visual working memory） |
|  | Benton Visual Retention Test |
|  | Rey Complex Figure Delayed Recall |
|  |  |
| Learning and memory | delayed recall from Logical Memory |
|  | Visual Reproduction |
|  | Auditory Verbal Learning Test |
|  | California Verbal Learning Test-II |
|  | RAVLT Trial 6 (Post-interference Recall Trial) |
|  | immediate and delayed recall using words from Alzheimer’s Disease Assessment Scale-cognitive( ADAS-Cog) |
|  |  |
| verbal fluency (part of executive function) | Phonemic fluency and Semantic fluency |
|  | RVR test |
|  | FAS Verbal Fluency Test |
|  | Letter Verbal Fluency Task(part of UHDRS) |
|  | COWAT Total Score |
|  |  |
| Language/naming | Graded Naming Test |
|  | Oldfield Naming Test |
|  | Boston Naming Test |
|  | Controlled Oral Word Association Test(COWAT) |
|  | Animal Naming Test |
|  | the Sydney Language Battery(Semantic and phonological processing) |
|  | the Token Test |
|  |  |
| Executive function | Wisconsin Card-Sorting Test (WCST) |
|  | finding words from switching semantic categories |
|  | Alzheimer’s Disease Assessment Scale-cognitive executive command |
|  | Stroop Test(behavioral self-regulation)A |
|  | Attentional matrices: selective attention and information processing speed test |
|  | Trail Making Test (TMT-B) |
|  | The tower of London(ToL) |
|  | recognition memory test for faces/words(RMTF/W) |
|  | Hayling Sentence Completion test(Hayling Test) |
|  | Stroop Colour-Word task(C-W Test) |
|  | the Weigl test(Weigl’s sorting test) |
|  | Circle-Tracing task(visuomotor integration and motor planning) |
|  | Conditional Association Task (CALT) |
|  | Behavioral Dyscontrol Scale( BDS) |
|  | Frontal Assessment Battery (FAB) |
|  | Design Fluency Delis Kaplan Executive Function System (DKEFS) |
|  | Addenbrooke’s Cognitive Examination Revised (ACE-R) |
|  |  |
| Psychomotor speed/information process speed | The Symbol Digit Modalities Test (SDMT) (part of UHDRS) |
|  | Stroop word reading (part of UHDRS) |
|  | Speeded Tapping–Thumbs, mean good responses |
|  | Serials 2’s Speeded Tapping task(maximum tapping) |
|  | Two choice reaction time |
|  | Cued movement sequencing |
|  | the processing speed index of WAIS-III(IV) |
|  | Symbol Counting Test |
|  | TMT-A |
|  | CAT(Clinical Assessment for Attention): Visual Cancellation task |
|  | Simple visual reaction time |
|  | Symbol Search (WAIS-IV) |
|  | Purdue Grooved Pegboard Test |
|  |  |
| Attention or working memory | Digit span forward(DSF, test for attention) and digit span backward(DSB, test for working memory)(part of WAIS-III) |
|  | Clinical Assessment for Attention(CAT) |
|  |  |
| Working memory | The Spatial Span (SSP) |
|  | The tower of London (ToL CPU) |
|  | Letter-Number Sequencing |
|  | Dual Verbal Working Memory Test |
|  | 2-back working memory |
|  | The Arithmetic subtest(a subset of WAIS-R, calculation) |
|  | Trial 1 of RAVLT(the Rey Auditory Verbal Learning Test) |
|  | WAIS-III Letter–Number Sequencing |
|  | WAIS–III Working Memory Index |
|  |  |
| Attention | the subtest Digit Span of the Revised Wechsler Memory Scale (WMS) with forward and backward reproduction being tested separately |
|  | Intra/ExtraDimensional (IED) Shift tests |
|  | WAIS–III Block Design Subtest(visuospatial function and visual attention) |
|  | The Test of Everyday Attention-Map Search Task(selective attention) |
|  | Symbol Counting Test(reflecting the processing speed) |
|  | Stroop Colour/Word |
|  | Trail Making Test B |
|  |  |
| Visuoperceptual and constructive functions  or visuospatial memory | Clock Drawing Test |
|  | Benton test (orientation of couple of lines) |
|  | Block design (WAIS-III) |
|  | Judgment of Line Orientation(JLO) |
|  | Hooper Visual Organization Test(HVOT) |
|  | Block Design and Object Assembly subtests from the WAIS-R(timed and untimed version) |
|  | VPTA: Copying Figures  VPTA: Bisection of Lines  VPTA: Copying Flowers  (Visual Perception Test for Agnosia) |
|  | Street’s completion test |
|  | the Visual Object and Spatial Perception battery |
|  | the Rey-Osterrieth Complex Figure Test (RCFT) |
|  | Forward Corsi span |
|  | Corsi’s blocks span tests |
|  |  |
| Sensory-perceptual ability | Benton Facial Recognition Test |
|  | Emotions Task–Static/dynamic Version |
|  | Emotion recognition |
|  | computerised presentations of photographs depicting six basic emotions or a neutral expression |
|  | Paced Tapping test（Psychomotor function）(sensory-perceptual) |
|  |  |
| Abstract reasoning or logical thinking | Raven’s Progressive (colored) Matrices (Raven PM) |
|  | subtest z Wechsler Memory Scale III, LM |
|  | The Test for Reception of Grammar, version 2 |
|  | Visual Perception Test for Agnosia( VPTA): Story Telling |
|  | WAIS-III: Similarities |
|  | Raven’s coloured progressive matrices(Visuospatial abstract reasoning) |
|  | Comprehensive Functional Evaluation-Solving Question Function-Logical Thinking Function |
|  |  |
| Orientation | Alzheimer’s Disease Assessment Scale-cognitive-Orientation |
|  |  |
| Calculation | the Graded Difficulty Arithmetic test |
|  | Arithmetical facts (eight single-digit operations) |
|  |  |
| Spelling | the Baxter Spelling tests |
